# Exploring the Mechanism of Microarteriogenesis in Porous Silk Fibroin Film

**DOI:** 10.1155/2012/262890

**Published:** 2012-12-05

**Authors:** Lun Bai, Guangqian Wang, Xiaoyan Tan, Jianmei Xu

**Affiliations:** ^1^School of Textile and Clothing Engineering, Soochow University, Suzhou 215021, China; ^2^Faculty of Textile Science and Technology, Shinshu University, Ueda 386-8567, Japan

## Abstract

*Purpose*. Based on the experiment of the microarteriogenesis that is associated with angiogenesis during tissue repair process in porous silk fibroin films (PSFFs), we investigate the characteristics of micro-arteriogenesis and explore its mechanism. *Methods*. After the porous silk fibroin materials are implanted into the back hypodermal tissue of SD rats, the arteriole development and the morphogenesis of smooth muscle cell are histologically monitored and the micro-arteriogenesis is quantitatively analyzed. *Results*. 10 days after implantation, the arteriole density reaches the highest level in the junction of silk fibroin materials with tissues. Three weeks later, the arteriolar density in the materials reaches the maximum, and the arterioles in the junction of materials with tissues appear to be in a mature and upgrading state. *Modeling of Microarteriogenesis*. The arterioles in materials are generated after capillary angiogenesis. It is inferred that arteriolar development does not start until the network of the capillaries is formed. At first, the arterioles grow in the conjunct area of precapillaries with arterioles. Then with the extension of the arterioles, the upgrade of arterioles in connecting area is observed at a later stage. Based on the observation, the conditions and the mechanism of microarterializations as well as the upgrade of arterioles are analyzed.

## 1. Introduction 

Within 10 days after implanting porous silk fibroin film (PSFF) into hypodermal tissue of rat, the formation of the capillary network system in materials was observed [1]. According to Bai et al. [2, 3], newly formed arterioles were found in materials not long after implanting PSFF into hypodermal tissue of rat, which shows that in the process of capillary formation, the arterioles also develop simultaneously. However, the relevant reports about the development of arterioles were seldom seen, although the arterioles play a major role in capillary network system. In recent years, there have been many studies on inducing the growth of collateral arteries to treat clogged arteries in clinical treatment [4–7]. The formation of arterioles in biological materials occurs in the circumstances where no blood vessels exist. Therefore, we can only imagine that the arteries are made by capillary transformed. As there is no smooth muscle in the periphery of capillary, so inducing the peripheral vascular smooth muscle cells to grow is the key of the transformation. Larouche and Schiffrin [8] reported that the arteriole formation is a result of the invasion of the smooth muscle cells into the capillaries under the impact of some unknown mechanisms. According to Fay Hansen-Smith et al. [9], arterioles grow as a result of the capillary arterialization, and the process is completed in two ways: first, the pericytes are transformed into smooth muscle cells; second, the interstitial cells (the vast majority may be fibroblasts) attached to the capillary wall surface gradually transform into pericytes or smooth muscle cells. As Skalak et al. [10] pointed out, in the case of chronic vasodilation some hemodynamic factors stimulate both the growth of capillaries and the transformation of capillaries into arterioles in skeletal muscles. However, the issue of the transformational mechanism of capillaries is not resolved. This paper focuses on the transformational process of capillaries into arterioles in biomaterials, including inducing causes and conditions, location and shape of arteriole formation. Based on experimental observation as well as analysis of arteriole formation, the study explores the cellular and molecular biological mechanism on the formation of arterioles in PSFFs. 

## 2. Materials and Methods

### 2.1. Preparation of PSFF

The sample of porous silk fibroin materials is prepared by adding certain specific additives to silk fibroin aqueous solution, which is then lyophilized under certain freeze-drying conditions. By controlling the structure of fibroin solution and freeze-drying conditions, the physical form parameters of the material, pore size, porosity, and so forth can be adjusted. The material samples are treated by irradiation sterilization. 

### 2.2. Implantation of PSFF

Sixteen healthy SD rats of SPF grade were selected for experiment. The rats were all between 250 g and 280 g in weight. During the operation, the rats were initially anesthetized with 2.5% pentobarbital sodium (35 mg kg^−1^). After the surgical site was sterilized with alcohol, a full skin (2 × 2 cm^2^) was cut off on the back of rat and partial dermis was removed. Then the prepared PSFF of about the same size was implanted under the dermis layer. Finally, the incision was sutured, the bleeding stopped, the wound cleaned, and the rats were brought back for normal feed.

### 2.3. Observation and Analysis of Sample

After the operation, the porous silk materials were sampled on the 5th, 7th, 10th, 13th, 15th, 18th, 23th, and 25th days. The samples were HEs stained for the histological examination and fastened with 10% formalin at the room temperature. Then paraffin-embedded sections were performed, HEs staining was completed, and resin was mounted, microscope-examined, and photographed. Finally the capillaries and arterioles in the photo were observed and statistically analyzed. In the microscope observation of tissue sections, for every slice 3 to 5 fields (×100) are randomly selected to be observed.

## 3. Results

### 3.1. The Microstructure of PSFF

**Figure 1:**
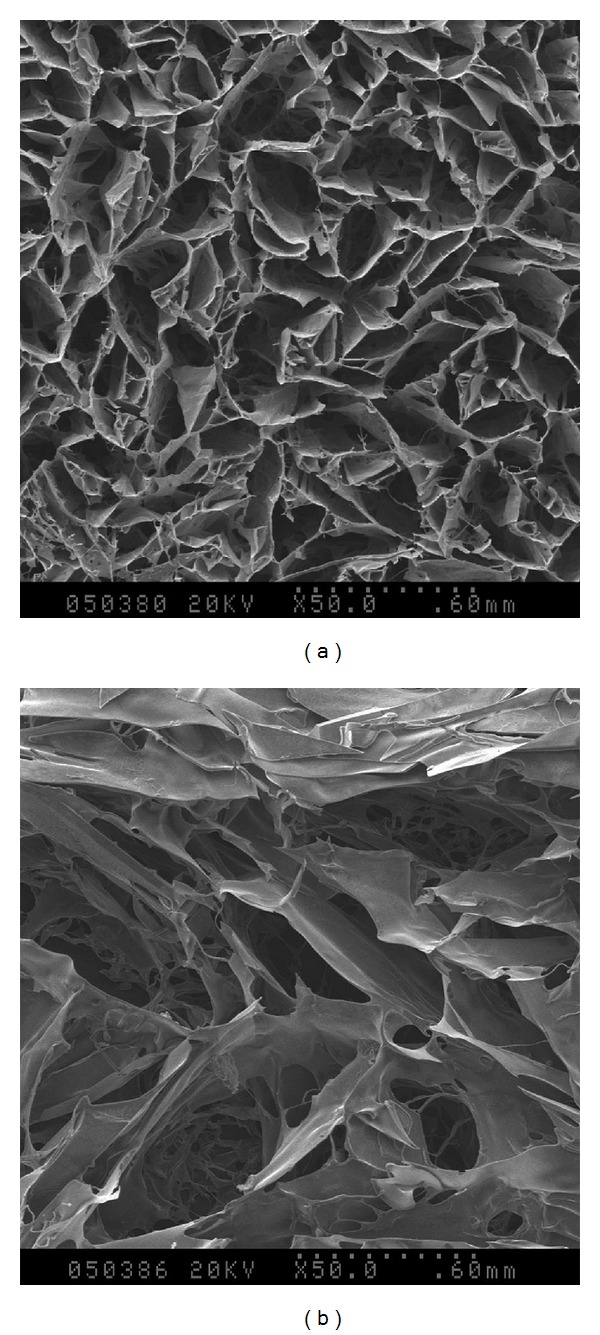
Macrostructure of PSFFs by SEM.

The average pore diameter of the silk fibroin film used in the experiment varies from 60 *μ*m to 80 *μ*m, and the porosity is about 90%. The size of the implanted film is about 2 × 2 cm^2^, with a thickness of about 0.8 mm.  Figure 1 shows the morphological structure of the PSFF.

### 3.2. Tissue Slice Observation of the Arteriolar Development

**Figure 2:**
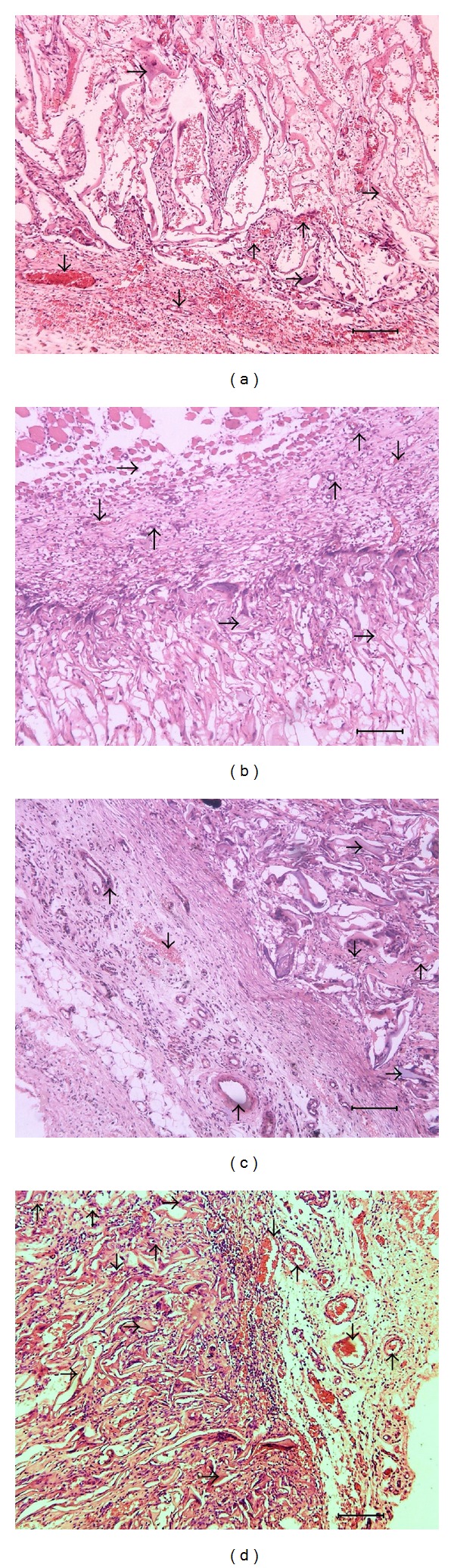
Tissue slices of PSFFs at different time (×100). (a, b, c, and d are the images at 5 d, 10 d, 18 d, and 23 d after implantation; ↓: venules ↑: arteriole; →: porous silk fibroin film; bar: 100 *μ*m).

**Figure 3:**
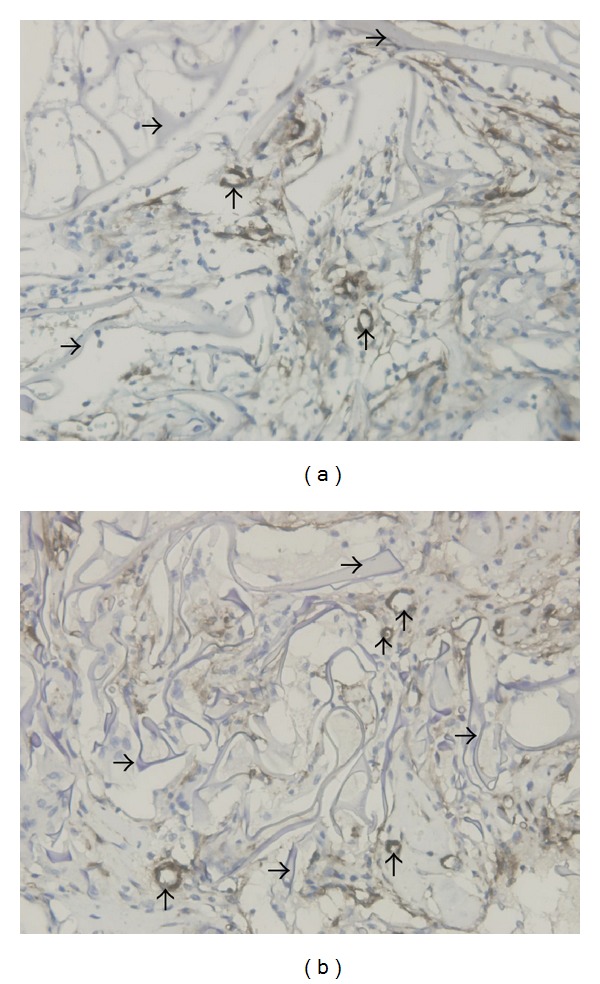
The images of HEs stained tissue (×400). (a, b are the images at 7 d, 20 d after implantation; ↑: arteriole; →: porous silk fibroin film).

Figure 2 is a group of photos of the arteriole formation in PSFF material. The microexamination of the tissue slices shows that 5 days after the surgery (Figure 2(a)), collagen fibers fill the pores of the material and many blood vessels, including arterioles, are observed around the periphery of the material. Red blood cells are easily found spreading around, and it is shown that the capillaries have grown into the superficial zone of the material, but no arterioles were found. No new vessels are found deep inside the material. Seven days after the surgery, the muscle connective tissue between tissues and materials continues to grow thicker, but the density of vessels decreases slightly. At the edge of the material, arterioles begin to show, but most are capillaries. According to the statistical analysis, the arteriolar density at the conjunct area is about 18–21 per mm^2^. On the 10th day after the survey, more arterioles are observed in the surrounding conjunct tissue (Figure 2(b)). The PSFF has partly degraded and become an integrated mass with the tissue. Thirteen days after the surgery, the arteriolar density in the conjunct tissue decreases, but is higher than that on the 7th day. After 15 days of the implantation, the arteriolar density continues to decrease in the conjunct tissue. On day 18 (Figure 2(c)), the arteriole density at the conjunct region between material and tissue is about 30–41 per mm^2^. On day 23, the PSFF has degraded obviously, the capillaries distributed in new tissue can be seen, and a relatively complete network of blood vessels seems to have been formed. At this time, arterioles of 150 *μ*m in diameter appear in the conjunct area (Figure 2(d)), showing the upgrading of arterioles and correspondingly some thick venules can also be observed. On the 25th day, the residual PSFF continues to degrade and no change is observed in the density of arterioles in conjunct area, and the microvascular density in PSFFs is roughly the same with surrounding tissue.  Figure 3 shows the images of HEs stained tissue implanted after (Figure 3(a)) 7 days and (Figure 3(b)) 20 days. In image (a), the arterioles can be seen in the tissue outside the materials, and in image (b) it is observed that the arterioles already formed inside the materials.

### 3.3. Quantitative Analysis of Arterioles

**Figure 4:**
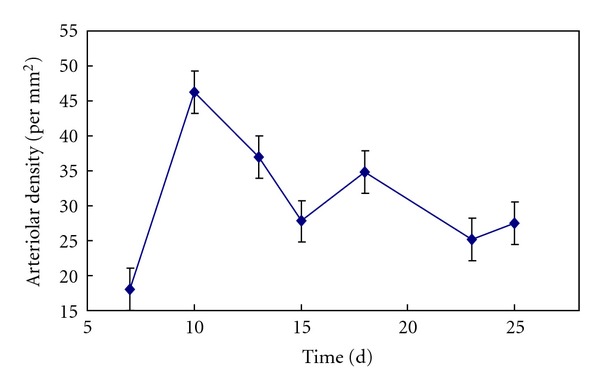
The curve of arteriolar density change with time in conjunct area between material and tissue.

**Figure 5:**
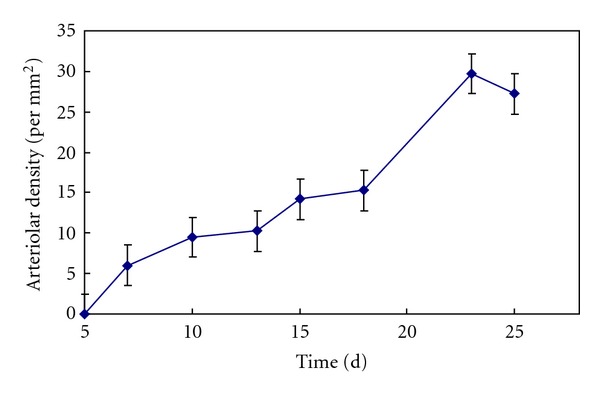
The curve of arteriolar density change with time in PSFFs.

Based on the statistical analysis, Figure 4 shows a curve of the arteriolar density change with the time after implantation in the conjunct area between material and tissue. As illustrated in the figure, the arteriolar density reaches the maximum value on the 10th day after the implantation. Then it begins to decrease and never returns to the peak, and tends to stabilize gradually although the pattern appears to be fluctuating.  Figure 5 illustrates a curve of the arteriolar density change with the time after implantation in PSFF materials. It can be seen that the arteriolar density is in an increasing state from the 7th day after the surgery and reaches the maximum on day 23, and then enters relatively stable state. Statistical results show that the arteriolar density in the materials reaches the maximum value in about 1 to 2 weeks later than the conjunct area between material and tissue. 

## 4. Discussion

A report [11] shows that the length of arterioles of a human being is 80 km and that of capillaries is 1200 km, which means that, on average, the ratio of capillary length to arteriole length is 15 : 1. Therefore, in the implanting experiment, it is clear that the arteriolar formation in material cannot be seen until sometime after capillaries begin to grow. In this section, the issues related to arteriolar formation in materials and its mechanism will be discussed. 

### 4.1. The Origin of Arteriole in Materials

The key of the arteriolar growth through the capillary transformation lies in the induced growth from smooth muscle cells. It is reported that the smooth muscle cells originate from the pericytes or interstitial cells which adhere to the wall of capillaries [9]. The experiment results indicate that arterioles grow first in the conjunct area between PSFFs and tissue, while the formation of arterioles inside the materials cannot be observed until sometime after capillaries grow inside the materials, which indicates that the arterioles are formed from the capillaries transformed. In order for the arterioles to continuously grow, it is necessary that the new arterioles are formed from the precapillaries connected with arterioles. Under the circumstances, when the smooth muscle cells develop in the periphery of capillaries, they may originally be derived from the pericytes and stromal cells [9], and another possible source to be considered is the smooth muscle cells adhered to the arterioles or precapillaries, which proliferate and extend constantly. In the experiment, in the connected area between tissues and material, where tissue metabolism is very active, the new arterioles develop and upgrade from original arterioles or precapillaries, the density and the diameter of the arteriole are larger than that inside the materials. This shows the continuity of the arteriolar growth. 

**Figure 6:**
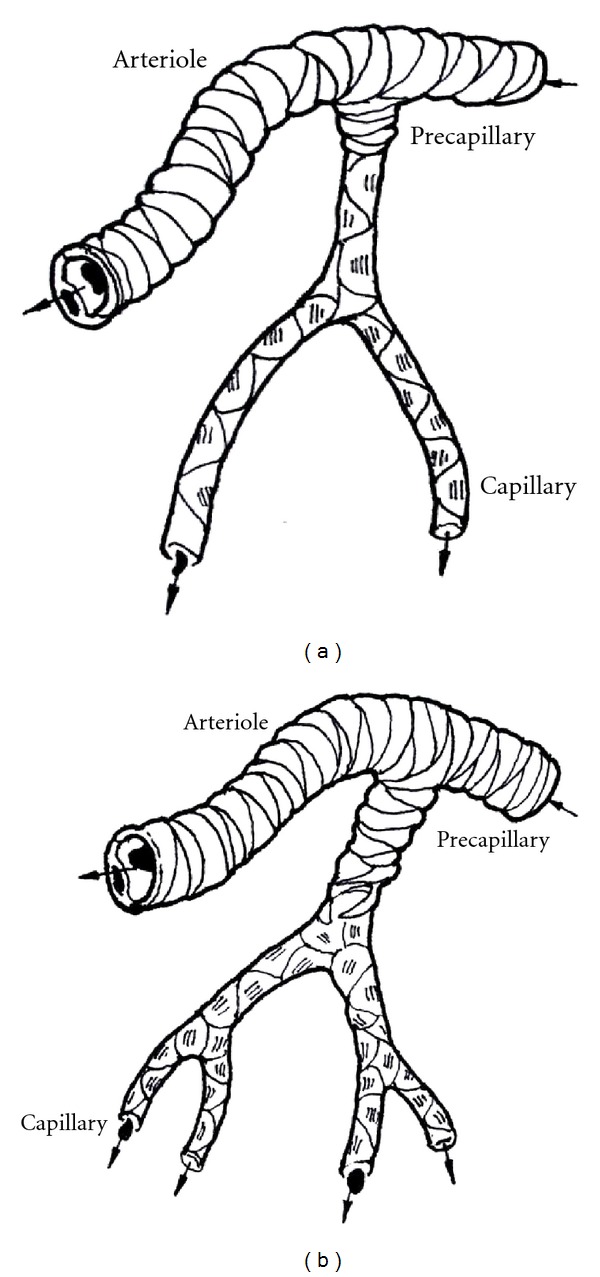
Illustration of precapillary transformation into arterioles. (a) Precapillaries before the increase of blood flow; (b) precapillaries after the expansion of capillary network.

In the angiogenesis process, once new capillaries form the loops and the capillary network develops to mature in PSFFs, the blood flow of the precapillaries between new capillary networks and arterioles will increase dramatically, which will extend the opening time of precapillaries. Therefore in the capillary networks the blood flow increase will cause vessel walls to expand, endothelial cells to extend, and cell permeability to increase. Because of the increment of the infusion time, the extending endothelial cells receive adequate supplies of oxygen and nutrients and speed up in proliferation, thus leading to the proliferation and migration of the smooth muscle attached to the wall of capillaries (Figure 6). Report [12] shows that when the endothelial cells in one end of rat aorta are stretched *in vitro* and then cultivated by 20% serum, the proliferation of the smooth muscle cells in vascular tunica media will occur. The experiment results support the idea that when the perfusion of blood flow in the expanded blood vessels increases, the smooth muscle cells will proliferate. When the smooth muscle cells proliferate and extend along the capillaries, due to the increased cell permeability the absorption efficiency of the endothelial cells on the oxygen and nutrient increases, resulting in endothelial cell proliferation to complement the needs for expansion of the vascular wall and causing the precapillaries to ultimately transform into microarterioles. At the same time, other precapillaries attached to the aboriginal arterioles will also transform into arterioles once the blood vessels expand to a certain extent. 

As a result, the blood flow of the aboriginal arteriole increases, the vascular endothelial cells continue to expand, and the blood vessels continue to suffuse for supplying blood flow, which leads to the proliferation and extension of the smooth muscle cells of aboriginal arterioles to adapt to the state of arteriolar persistent suffuse. At the same time, the arteriolar diameters increase and the arterioles overall upgrade. Therefore, after the implantation of PSFFs into the tissue, the capillary network developed after angiogenesis will cause some precapillaries to transform into arterioles, so that it leads the aboriginal arterioles to upgrade, the smooth muscle cells to proliferate, the vascular wall to thicken, and the vascular diameter to increase. This process starts from the downstream of capillary network. In the experiment, it can be seen that, with the capillaries gradually getting into the inner material, the arterioles gradually grow into the inner material from the peripheral fringe of materials. 

### 4.2. The Precapillaries Induced to Arterioles

In the initial stage after the implantation of PSFF, with the gradual entry of tissue fluid and cells, the pore space of the material is in the continuous hypoxic state because of the tissue trauma and the active tissue response in the repair process. Angiogenesis is triggered by the initial strong hypoxia state [1, 13]. Once the network of capillary is formed, the new capillary network will assume the function of oxygen supply for damage repair and metabolism of new tissues. In the environment of strong metabolism, the closed period of producing the metabolic wastes becomes short in capillary units, and the opening period of capillaries relatively is extended [14], which makes the proportion of the open capillaries in tissue area increase. Meanwhile, the frequency of exchanging the metabolites (O_2_, nutrients and CO_2_, wastes), that is, the change of the states (open or close) of capillary increases [14]. The lengthing of the opening period of precapillaries means that the endothelial cells are often in the extended state, which enables the endothelial cells and their surrounding tissues to get adequate supply of oxygen and nutrients from the suffusing blood flow. As mentioned earlier, this will cause the smooth muscle (sphincter) cells on the precapillaries to extend and proliferate, and the vascular wall to become thicker and the precapillaries to transform into arterioles.

### 4.3. The Upgrading Mechanism of Capillary and Arteriole

Based on the discussion above, the conditions that the capillaries and arterioles are upgraded to the higher level of blood vessels can be summarized as follows: (1) the vascular walls exist in the stretching state (so far reported as shear force) for a long time, thus causing the endothelial cells to extend, which enables the cell permeability to improve; (2) due to the relatively prolonged perfusion time (for capillaries) or increased traffic flow and increased flow speed, endothelial cells in the expansion state are able to receive even greater supplies of oxygen and nutrients. As a result, its effects are spread to the proliferation of the smooth muscle cells attached to the outer periphery of precapillaries and to the transformation of the pericytes into the smooth muscle cells. When these two conditions are met, it will cause a series of the physiological regulations, such as signal activation and signal transduction and microenvironmental changes, thus promoting cell proliferation and vascular restructuring, making it possible that the precapillaries and arterioles are upgraded to a higher vascular level. Further issues in molecular level are not discussed in this paper. Since the formation of arterioles is based on the existing capillary network, after the emergence of new capillary networks in the biological materials, it is possible that arterioles are developed in the pores of materials.
